# Recruitment of Activation Receptors at Inhibitory NK Cell Immune Synapses

**DOI:** 10.1371/journal.pone.0003278

**Published:** 2008-09-26

**Authors:** Nicolas Schleinitz, Michael E. March, Eric O. Long

**Affiliations:** Laboratory of Immunogenetics, National Institute of Allergy and Infectious Diseases, National Institutes of Health, Rockville, Maryland, United States of America; Centre de Recherche Public-Santé, Luxembourg

## Abstract

Natural killer (NK) cell activation receptors accumulate by an actin-dependent process at cytotoxic immune synapses where they provide synergistic signals that trigger NK cell effector functions. In contrast, NK cell inhibitory receptors, including members of the MHC class I-specific killer cell Ig-like receptor (KIR) family, accumulate at inhibitory immune synapses, block actin dynamics, and prevent actin-dependent phosphorylation of activation receptors. Therefore, one would predict inhibition of actin-dependent accumulation of activation receptors when inhibitory receptors are engaged. By confocal imaging of primary human NK cells in contact with target cells expressing physiological ligands of NK cell receptors, we show here that this prediction is incorrect. Target cells included a human cell line and transfected *Drosophila* insect cells that expressed ligands of NK cell activation receptors in combination with an MHC class I ligand of inhibitory KIR. The two NK cell activation receptors CD2 and 2B4 accumulated and co-localized with KIR at inhibitory immune synapses. In fact, KIR promoted CD2 and 2B4 clustering, as CD2 and 2B4 accumulated more efficiently at inhibitory synapses. In contrast, accumulation of KIR and of activation receptors at inhibitory synapses correlated with reduced density of the integrin LFA-1. These results imply that inhibitory KIR does not prevent CD2 and 2B4 signaling by blocking their accumulation at NK cell immune synapses, but by blocking their ability to signal within inhibitory synapses.

## Introduction

Natural killer (NK) cells express a variety of inhibitory receptors that recognize MHC class I molecules and block NK cell–mediated cytotoxicity [1], [2]. In human NK cells, these receptors include the Killer cell Ig-like Receptor (KIR) family, the Leukocyte Immunoglobulin-like Receptor (LILR) family, NKR-P1, and the family of CD94/NKG2 lectin-like receptors. Phosphorylated immunoreceptor tyrosine–based inhibition motifs (ITIM) in the cytoplasmic tails of such inhibitory receptors recruit the tyrosine phosphatases SHP-1 and SHP-2 [3]–[5]. Inhibition occurs through SHP-mediated dephosphorylation of key components in the signaling pathway for activation, such as Vav1 [6]. Inhibition by KIR blocks NK cell activation at a very proximal step, which precedes actin-dependent processes [7]. For instance, binding of inhibitory KIR to MHC class I on target cells prevents the tyrosine phosphorylation of activation receptors 2B4 and NKG2D, as well as their recruitment to detergent-resistant membrane microdomains [8], [9]. Engagement of ITIM-containing inhibitory receptors blocks the accumulation of F-actin at T cell and NK cell immune synapses [10]–[12], and prevents the actin-dependent accumulation of glycosphingolipid-enriched domains at inhibitory synapses in YTS cells [13] and NK clones [9], [14].

Reorganization of the actin cytoskeleton is essential for the cytotoxic activity of T cells and NK cells. Inhibitors of actin polymerization prevent cytolytic activity, hinder accumulation of receptors at activating immune synapses [15], and block phosphorylation of NK cell activation receptors [8], [9]. Given that actin cytoskeleton rearrangement is inhibited by ITIM-containing receptors, it is generally assumed that KIR engagement at an inhibitory synapse prevents the delivery of activation signals by blocking the cytoskeleton–dependent movement of activating receptors. To test this hypothesis, we visualized the distribution of activation receptors 2B4 and CD2 in activating and inhibitory NK cell immune synapses, using primary human NK cells. We report the surprising finding that KIR engagement at inhibitory synapses promotes the accumulation of activation receptors 2B4 and CD2.

## Results

### Detection of activating and inhibitory immune synapses between target cells and primary human NK cells

We wished to study NK cell immune synapses in unmanipulated, polyclonal human NK cells in order to avoid complications or biases that may arise in the cloning of NK cells or the expression of exogenous proteins in NK cells. To do this, it was necessary to identify NK cells expressing the receptors of interest. All human NK cells express the β2 integrin LFA-1 and activation receptor 2B4, whereas a subset of NK cells express CD2. Expression of MHC class I-specific inhibitory receptors, including KIR, on NK cells is more complex. KIRs are clonally distributed among NK cells, with any given NK cell expressing its own KIR repertoire. Furthermore, monoclonal Abs to KIRs do not distinguish between inhibitory KIR2DL1 and the short–tailed, activating KIR2DS1, or between inhibitory KIR2DL2 and activating KIR2DS2. To identify inhibitory KIR2DL, a polyclonal antiserum against the conserved C-terminal amino acids of KIR2DL1 and KIR2DL2 was raised (cyt42/43 antiserum). The short cytoplasmic tails of KIR2DS1 and KIR2DS2 do not include the amino acid sequence reactive with cyt42/43 antiserum. Inhibitory synapses were identified by the clustering of KIR2DL1 towards target cells expressing its ligands HLA-Cw4 or HLA-Cw15, and by the clustering of KIR2DL2 towards target cells expressing its ligand HLA-Cw3. Several controls were performed to validate this approach.

**Figure 1:**
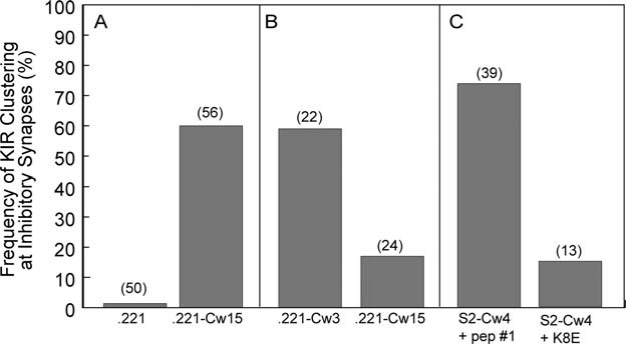
Detection of inhibitory synapses using the cyt42/43 antiserum. IL-2 activated polyclonal human NK cells were mixed with target cells for 10 minutes at 37°C, fixed, permeabilized and stained with the cyt42/43 rabbit polyclonal antiserum. NK cells and NK:target cell conjugates stained with the cyt42/43 antiserum were scored for KIR clustering. The number of cyt42/43-positive cell conjugates analyzed is given in parentheses over each bar. (A) Mixing with .221 and .221-Cw15 target cells, as indicated. (B) NK cells expressing KIR2DL2 and not KIR2DL1 mixed with .221-Cw3 and .221-Cw15 cells, as indicated. (C) Mixing with S2–Cw4 cells loaded with a peptide that is permissive for KIR2DL1 binding (peptide #1) or a peptide that is nonpermissive for KIR2DL1 binding (K8E), as indicated.

Primary NK cells were incubated with HLA class I-negative 721.221 cells, and 721.221 cells expressing HLA-Cw15. Cell conjugates were allowed to settle onto poly-L-lysine–coated coverslips, fixed, permeabilized, and stained for CD11a and inhibitory KIR2DL1/KIR2DL2. No clustering was detected in NK cells in contact with 721.221 cells, whereas 60% of cyt42/43-positive cells in contact with 221-Cw15 cells displayed clustered KIR (Fig 1A). Therefore, KIR2DL1 clustering occurs in at least 60% of KIR2DL1–positive NK cells. An NK cell population from a donor that expressed KIR2DL2 but not KIR2DL1 was also tested. In such NK cells, all of the cyt42/43-reactivity is directed at KIR2DL2. Sixty percent of KIR2DL2–expressing cells in contact with 221-Cw3 cells displayed inhibitory KIR clustering (Fig 1B). 17% of KIR2DL2^+^ NK cells formed clusters with 221-Cw15 cells (Fig 1B), which could be explained by the known crossreactivity of KIR2DL2 with HLA-Cw15 [16], [17].

KIR clustering was also assessed with *Drosophila* S2 cells that express HLA-Cw4, a ligand of KIR2DL1. In this system, the target cells express well-defined combinations of ligands for human NK cell receptors. As shown previously, expression of peptide-loaded HLA-Cw4 on insect cells is sufficient to induce clustering of KIR on NK cells [18]. As insect cells cannot load peptides, HLA class I reaches the cell surface “empty” [19]. Clustering of inhibitory KIR was observed when HLA-Cw4 on S2 cells was loaded with a peptide that is permissive for KIR2DL1 binding [20] (Fig 1C). For comparison, HLA-Cw4 was also loaded with a peptide that is not permissive for KIR2DL1 binding [20]. In this case, KIR2DL1 clustering was much less frequent (Fig 1C). For the purpose of this study, immune synapses were considered inhibitory when conjugates between HLA-Cw4 or HLA-Cw15-expressing target cells and NK cells displayed clustering of cyt42/43-reactive KIR. NK cells in conjugates that lacked cyt42/43 reactivity altogether (i.e. negative for KIR2DL1 and KIR2DL2) were scored as activating immune synapses. By this approach, it was possible to distinguish activating and inhibitory immune synapses within the same population of primary NK cells that were in contact with target cells expressing well-defined ligands of NK cell activating and inhibitory receptors.

### Activation receptors CD2 and 2B4 accumulate at inhibitory immune synapses

The distribution of most activation receptors at inhibitory NK cell immune synapses has not been examined. Accumulation of CD2 at activating immune synapses is dependent on the protein WASp and actin polymerization [15]. Rapid accumulation of 2B4 at activating immune synapses has been visualized in live cells [21]. The phosphorylation and recruitment of 2B4 to detergent-resistant membrane domains, which are also dependent on actin polymerization, are blocked by co-engagement of inhibitory KIR [8]. As inhibitory ITIM–containing receptors prevent actin cytoskeleton rearrangement [10]–[12] and localization of GM-1–containing lipid rafts to NK cell immune synapses [13], one would expect inhibition of the actin polymerization–dependent clustering of activation receptors CD2 and 2B4 by KIR. Here, we determined the localization of receptors CD2 and 2B4 in both activating and inhibitory NK cell immune synapses.

**Figure 2:**
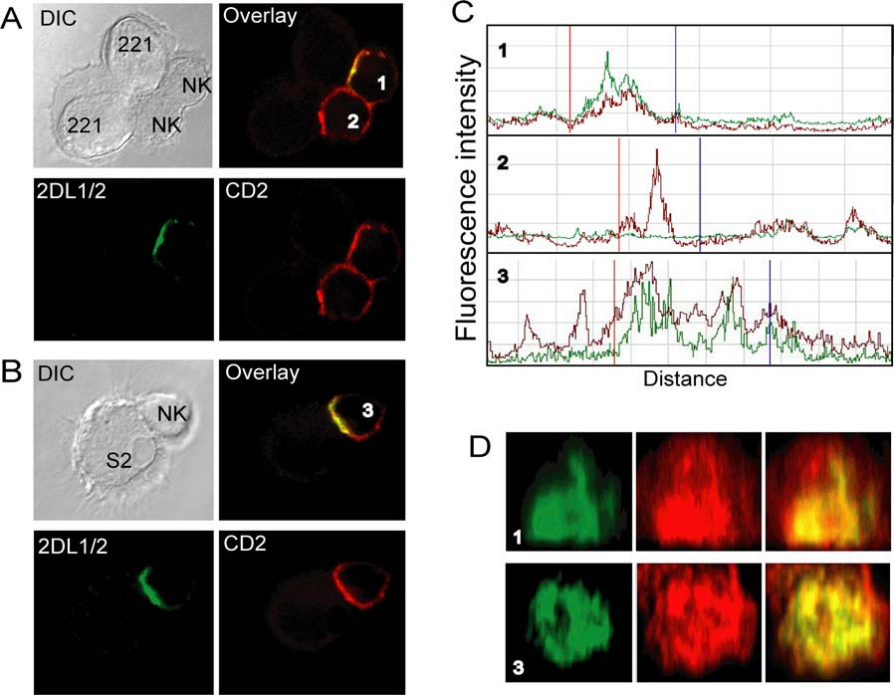
CD2 accumulates at both activating and inhibitory synapses. IL-2 activated polyclonal human NK cells were mixed with target cells at 37°C for 10 minutes, fixed, permeabilized, and stained with the cyt42/43 antiserum and a mAb to CD2 followed by the relevant secondary antibodies. Confocal microscope *z*-series were obtained. (A) Mixed with .221-Cw15 as indicated. A single confocal section is shown. The cell labeled #1, which displays KIR expression and clustering, represents an inhibitory synapse while cell #2, which lacks KIR2DL1 expression, displays an activating synapse. (B) Mixed with S2–LFA-3/Cw4 target cells, as indicated. A single confocal section is shown. (C) The fluorescence intensity was scanned around the perimeter of conjugated NK cells. Profiles labeled 1, 2, and 3 are from the corresponding cells in Figures 2A and 2B. The green and red lines represent the cyt42/43 and anti–CD2 fluorescence, respectively. Vertical red and blue lines mark the boundaries of cell contact as determined in DIC images. (D) Confocal *z*-stacks were used to create an *en face* view of the zone of cell contact in 2 inhibitory synapses.

**Figure 3:**
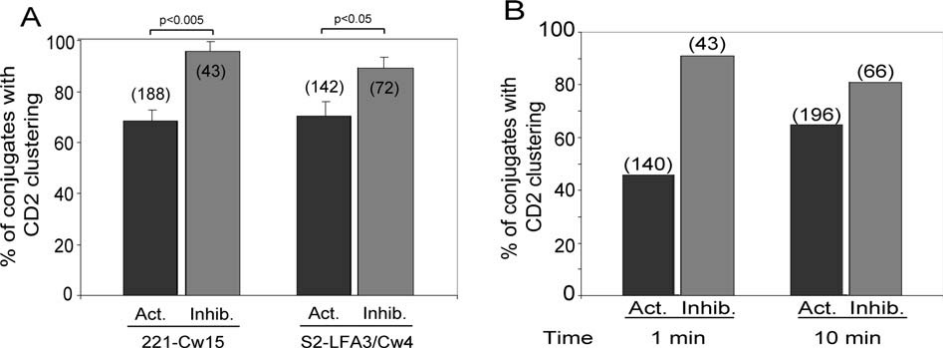
CD2 accumulates more frequently at inhibitory synapses than at activating synapses. (A) Conjugates between IL-2 activated polyclonal human NK cells and 721.221-Cw15 cells or S2–LFA-3/Cw4 cells were formed and stained as in Figure 2. Activating (Act.) and inhibitory (Inhib.) synapses were scored for clustering of CD2 at the zone of contact. The number of conjugates scored in each condition is indicated in parentheses. (B) Conjugates between activated NK cells and 721.221-Cw15 cells were allowed to form for 1 minute or 10 minutes as indicated, stained with the cyt42/43 antiserum and an anti-CD2 antibody as in Figure 2, and scored for CD2 clustering.

The MHC class I–deficient cell line 721.221 expresses LFA-3 and CD48, which are ligands for CD2 and 2B4, respectively. 721.221 cells transfected with HLA-Cw15 (221-Cw15), which is a ligand for KIR2DL1, were used as targets to evaluate the distribution of CD2 and 2B4 in NK cell immune synapses. Contrary to expectations, CD2 accumulated at both activating (Figure 2A, 2C, cell #2) and inhibitory (Figure 2A, 2C, cell #1) NK cell immune synapses with 221-Cw15 target cells. Reconstruction of the zone of contact in inhibitory NK–target cell inhibitory synapses showed that the intensity of CD2 staining correlated well with the intensity of KIR2DL1 staining (Figure 2D, cell #1). Similar results were obtained with S2 insect cells expressing LFA-3 and HLA-Cw4 (Figure 2B, 2C, 2D, cell #3), indicating that engagement of other receptors is not required for CD2 accumulation at NK cell immune synapses. Surprisingly, the frequency of CD2 clustering in NK cell conjugates with 721.221 cells and transfected S2 cells was higher at inhibitory immune synapses than at activating synapses (Figure 3A). Accumulation of KIR at inhibitory synapses is very rapid [12], [18]. To test whether KIR clustering may accelerate the accumulation of CD2, NK–target cell conjugates were allowed to form for only one minute. In contrast to activating immune synapses, in which CD2 accumulation was more limited at one minute, CD2 accumulation in inhibitory synapses at one minute was already as high as its accumulation at 10 minutes (Figure 3B). Therefore, KIR engagement with HLA class I on target cells promotes rapid accumulation of CD2 at inhibitory NK cell immune synapses.

**Figure 4:**
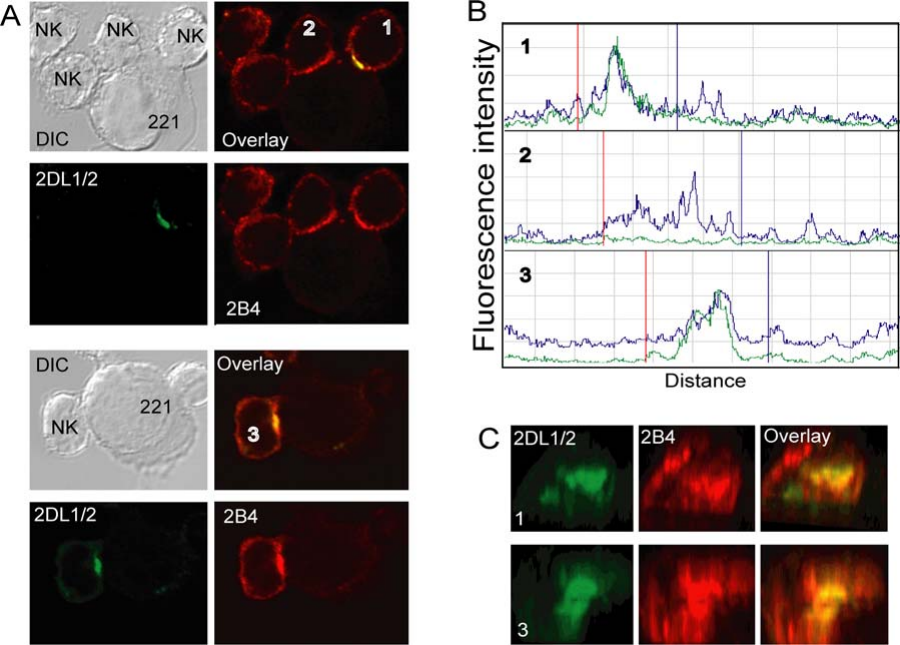
2B4 accumulates at both activating and inhibitory synapses. Activated NK cells were mixed with target cells at 37°C for 10 minutes, fixed, permeabilized, and stained with the cyt42/43 antiserum and a mAb to 2B4 followed by the appropriate secondary antibodies. (A) Mixed with .221-Cw15 target cells. Confocal microscope *z*-series were obtained, and single sections are shown. The cells labeled #1 and #3, which display KIR expression and clustering, represent inhibitory synapses while cell #2, which lacks KIR2DL1 expression, is an activating synapse. The NK cells without a number in the top image were not analyzed, because they did not appear to form tight conjugates with target cells. (B) The fluorescence intensity was scanned around the perimeter of conjugated NK cells. Profiles labeled 1, 2, and 3 are from the corresponding cells in Figure 4A. The green and blue lines represent the cyt42/43 and anti–2B4 fluorescence, respectively. Vertical red and blue lines mark the boundaries of cell contact as determined in DIC images. (C) Confocal *z*-stacks were used to create an *en face* view of the zone of cell contact in 2 inhibitory synapses.

Accumulation of activation receptor 2B4 was also observed in NK cells that formed activating (Figure 4A, 4B, cell #2) and inhibitory (Figure 4A, 4B, cells #1 and #3) immune synapses with 221-Cw15 cells. Three-dimensional reconstruction of the contact zone at inhibitory synapses showed that the intensity of 2B4 staining correlated well with the intensity of KIR staining, implying that, similar to CD2, 2B4 colocalizes with KIR at inhibitory NK cell immune synapses (Figure 4C).

### Exclusion of LFA-1 at inhibitory immune synapses

**Figure 5:**
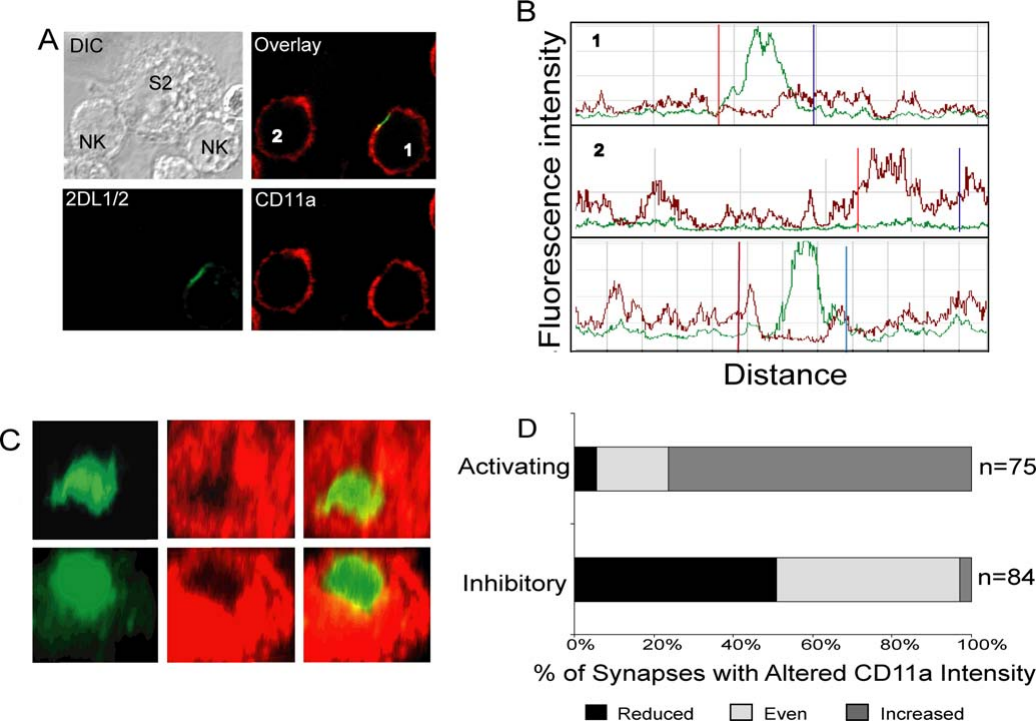
Surface level of CD11a is reduced at inhibitory synapses. IL-2 activated polyclonal human NK cells and S2 cells expressing ICAM-1 and peptide-loaded HLA-Cw4 were mixed at 37°C for 10 minutes, fixed, permeabilized, and stained with cyt42/43 and a mAb to CD11a followed by the appropriate secondary antibodies. (A) Confocal microscope *z*-series were obtained, and single sections are shown. The cell labeled #1, which shows KIR expression and clustering, represents an inhibitory synapse while cell #2, which lacks KIR2DL1 expression, displays an activating synapse. (B) The fluorescence intensity was scanned around the perimeter of conjugated NK cells. Profiles labeled 1 and 2 are from the corresponding cells in Figure 5A. The third profile is another representative inhibitory synapse. The green and red lines represent the cyt42/43 and anti–CD11a fluorescence, respectively. Vertical red and blue lines mark the boundaries of cell contact as determined in DIC images. (C) Confocal *z*-stacks were used to create an *en face* view of the zone of cell contact in 2 inhibitory synapses. (D) The frequency of synapses displaying increased CD11a, reduced CD11a, or no change in CD11a intensity was determined for both activating and inhibitory synapses.

Although inhibitory KIR segregate from LFA-1 at inhibitory synapses, different LFA-1 and KIR distribution patterns have been reported, which could be due to different levels of HLA-C expression on target cells [14], [22]–[24]. *Drosophila* S2 cells expressing ICAM-1 and HLA-Cw4 were used to examine the distribution of LFA-1 and KIR2DL1 in the absence of the many other receptor–ligand interactions that occur between NK cells and mammalian target cells. LFA-1 in NK–S2 cell conjugates was detected with an anti-CD11a antibody. Confocal *z*-series of activating and inhibitory synapses were acquired. CD11a staining on the NK cells was often uneven, with patches of CD11a around the cell periphery. However, careful quantitation of CD11a fluorescence on NK cells at activating synapses (Figure 5A, cell #2) revealed a reproducible increase of CD11a at the site of cell–cell contact, when compared to the rest of the NK cell membrane (Figure 5B, cell #2). Analysis of a number of conjugates showed that CD11a accumulates at approximately 75% of activating NK cell synapses (Figure 5D).

In contrast to the CD11a accumulation seen at activating synapses, CD11a at inhibitory synapses was reduced in the central zone of cell–cell contact (Figure 5A, 5B, cell #1). The overall distribution of LFA-1 on NK cells engaged in inhibitory synapses showed that KIR binding to HLA-C on target cells not only prevents accumulation of LFA-1, but actively excludes some of the LFA-1 from inhibitory synapses. Three–dimensional reconstruction of confocal z-stacks of inhibitory synapses revealed an obvious hole in the CD11a staining, which corresponded to the most intense KIR staining (Figure 5C). Quantitation of a number of conjugates showed that exclusion of CD11a from the zone of contact was observed in about half of the inhibitory synapse (Figure 5D). The other half of inhibitory synapses showed mostly unchanged CD11a fluorescence across the area of cell contact (Fig 5D). Therefore, the segregation of inhibitory KIR and LFA-1, which occurs at inhibitory synapses between NK cells and mammalian target cells, does also occur with insect cells expressing only ICAM-1 and HLA-C. In conclusion, the distribution of LFA-1 at NK cell immune synapses differs from that of activation receptors 2B4 and CD2. First, the extent of LFA-1 accumulation at activating immune synapses is more limited. Second, in contrast to activation receptors 2B4 and CD2, which accumulate at inhibitory synapses, LFA-1 is often excluded from the zone of KIR clustering.

## Discussion

The clustering of receptors that occurs upon ligand binding at cell–cell contacts is usually an energy-dependent process, the basis of which is still poorly understood [25]. Inhibition of actin polymerization blocks the accumulation of receptors CD2 and 2B4 at NK cell immune synapses, and the recruitment of 2B4 to detergent-resistant membrane domains [15], [26]. In contrast, inhibitory KIRs have the very unusual property of clustering independently of actin polymerization and of ATP metabolism when binding to an HLA class I ligand on target cells [22]. Expression of a cognate HLA-C ligand on transfected *Drosophila* cells was sufficient to induce KIR clustering [18]. As ITIM-containing inhibitory receptors prevent actin dynamics [10], [11], it was predicted that KIR inhibitory signaling would prevent the energy- and actin-dependent clustering of activating receptors, thereby blocking activation of NK cells. Indeed, clustering of the activation receptor NKG2D is inhibited by KIR engagement [9]. However, we report here the unexpected accumulation of activation receptors CD2 and 2B4 at both activating and inhibitory NK cell immune synapses. CD2 clustered at inhibitory synapses even more frequently and more rapidly than at activating synapses. The sensitivity to cytochalasin D (an inhibitor of actin polymerization) and to azide (an inhibitor of cytochrome c oxidase) of CD2 and 2B4 clustering was in fact lifted by KIR co-engagement (data not shown). We conclude that KIR does not inhibit, but rather promotes accumulation of CD2 and 2B4 at inhibitory immune synapses.

We have examined the clustering of receptors at activating and inhibitory synapses of primary, unmanipulated NK cells with target cells. To work with primary NK cells, one has to overcome the complication due to the heterogeneous expression of the family of inhibitory receptors, which are essentially randomly distributed on NK cells. To do so, inhibitory KIR2DL1 and KIR2DL2 were visualized in polyclonal NK cell populations with a specific antiserum raised against their conserved cytoplasmic tail. By this approach, inhibitory NK cell immune synapses were identified not only on the basis of the NK and target cell KIR and HLA phenotypes, but also by visible clustering of inhibitory KIR. In addition to the use of human target cells, which express ligands for many different NK cell receptors, experiments were also performed with transfected *Drosophila* insect cells. By expression of only one or a few ligands for human NK cell receptors, this insect cell system is ideally suited to dissect the contribution of individual receptors to NK cell activation [27], [28]. Furthermore, transfected *Drosophila* cells provide a very stringent test for MHC class I-dependent function. As MHC class I folding at the surface of insect cells requires addition of specific exogenous peptide [19], insect cells expressing MHC class I in the absence of peptide provide an ideal negative control. Using this system, we have shown that clustering of KIR correlated with reduced LFA-1 at the synapse, but did not prevent accumulation of CD2 or 2B4.

The reason for the unexpected coclustering of CD2 and 2B4 with inhibitory KIR may be to facilitate inhibition by maintaining proximity of activating receptors with the tyrosine phosphatases SHP-1 and SHP-2 recruited by KIR, thus providing the opportunity for rapid deactivation of signaling initiated by CD2 or 2B4. Otherwise, 2B4 and CD2 accumulation in a region peripheral to the clusters of KIR could be dangerous, as signaling could proceed unimpeded by KIR-dependent inhibition. The predicted sizes of KIR2DL1/HLA-Cw4, 2B4/CD48, and CD2/LFA-3 complexes are very similar, and much smaller than LFA-1/ICAM-1 complexes. Co-clustering of 2B4, CD2, and KIR2DL1, as well as the exclusion of LFA-1 from these clusters, could therefore result from partitioning based on size, as has been proposed for the T cell synapse [29].

A recent report visualized inhibitory signaling by KIR at NK cell immune synapses by detecting phosphorylation of the KIR ITIMs through fluorescence resonance energy transfer (FRET) imaging [30]. Surprisingly, KIR phosphorylation does not occur uniformly across the inhibitory synapse but in small clusters, suggesting that inhibition may be transient and local. In such a case, colocalization of inhibitory KIR with clusters of activation receptors would greatly improve KIR-dependent inhibition. Alternatively, clusters of phosphorylated KIR may represent KIR molecules that have been phosphorylated due to their proximity to signaling clusters of activation receptors. However, the fact that KIR prevents tyrosine phosphorylation of 2B4 and movement of 2B4 into detergent-resistant membrane domains, suggests that KIR phosphorylation is not downstream of 2B4 signaling [7], [26]. KIR prevents the recruitment of 2B4 and of NKG2D to detergent-resistant membrane domains [8], [9], and inhibits the accumulation of ganglioside GM1 at the synapse [9], [13], [14]. These observations suggest a model in which signaling by activation receptors is inhibited due to the failure of lipid raft-associated signaling molecules to coalesce at inhibitory synapses. The precise mechanism by which KIR inhibits NK cell activation will only be understood through the study of the unique biophysical properties of KIR, which lead to its unusual, energy- and actin-independent clustering.

## Materials and Methods

### Cells and Abs

Human NK cells were isolated from peripheral blood of healthy donors using an NK cell isolation kit (Miltenyi Biotech, Auburn, CA). NK cell purity was assessed by FACS and the cells were over 98% CD3^−^CD56^+^CD16^+^. IL-2 activated NK cells were cultured as previously described [27]. IL-2 activated NK cells were used for assays between 2 and 4 weeks after isolation. The 721.221 EBV-transformed cell line, as well as the transfectants 721.221-HLA-Cw15 and 721.221-HLA-Cw3 were maintained in Iscove's media supplemented with 10% fetal calf serum and L-glutamine. Drosophila Schneider Cell 2 (S2) cells were maintained as previously described [27]. Expression of transfected proteins was induced with 1 mM copper sulfate for 48 hours. For expression of HLA-Cw4 on S2 cells, either a peptide permissive for KIR2DL1 binding (peptide #1: QYDDAVYKL) or a non-permissive (K8E: QYDDAVYEL) was added at 1 µM during the last 20 hours of the induction. The following PE-conjugated antibodies were used for S2 cell staining: anti-HLA ABC (clone G46-2.6), anti-LFA-3 (1C3), anti-CD48 (HM48-1), and anti-CD54 (HA58) (Pharmingen, San Diego CA). For confocal imaging, the following primary antibodies were used (all from Pharmingen): anti-CD2 (RPA-2.10), anti-CD11a (HI111), and anti-2B4 (2–69), For detection of KIR2DL1 and KIR2DL2,a rabbit antiserum raised against the C–terminal sequence of the cytoplasmic tail of KIR2DL1 and KIR2DL2 (cyt42/43) was used.

### Conjugate Formation

IL-2 activated NK cells were resuspended with 10^6^ target cells at a 1∶1 ratio. The cells were gently vortexed, centrifuged at 300 RPM for 3 minutes at 4°C, and placed at 37°C for 10 minutes to allow conjugate formation. In the experiment shown in Figure 3B, cells were additionally allowed to form conjugates for only 1 minute. The cell pellet was directly chilled on ice for 10 minutes, and then cells were gently resuspended and allowed to settle on poly-L-lysine (Sigma, St. Louis, MO) coated coverslips for 15 minutes at 4°C. Cells were fixed in 4% paraformaldehyde. Cells were permeabilized in 0.5% Triton-X100 and 10% normal donkey serum (NDS) in PBS for 30 minutes at room temperature. Cells were stained with combinations of the following primary antibodies: 5 µg/ml anti-CD2, 0.1 µg/ml anti-CD11a, 12.5 µg/ml anti-2B4, and 5 µg/ml cyt42/43. Primary antibodies were diluted in 0.5% Triton-X100 and 3% NDS in PBS and incubated with the cells for 1 hour at room temperature. After three washes in PBS, cells were incubated for 1 hour at room temperature with the appropriate secondary antibodies in 0.5% Triton-X100, 3% NDS in PBS. Secondary antibodies used were Alexa 488–conjugated goat anti–mouse and goat anti–rabbit IgG (1∶2000 dilution), and Alexa 568–conjugated goat-anti mouse and goat-anti rabbit IgG (1∶1000 dilution) (Molecular Probes, Eugene OR). After three washes in PBS, coverslips were mounted to slides using the Prolong anti-fade kit (Molecular Probes).

### Confocal Microscopy

Images of stained conjugates were collected on a Zeiss LSM510 Meta confocal microscope system using a plan apochromat 63x/1.4 oil immersion objective. Excitation wavelengths used were 488nm (argon/krypton) and 543 (helium/neon). Differential interference contrast (DIC) images were collected simultaneously with the fluorescent images. Multitrack acquisition mode was used to avoid crosstalk between the different fluorophores. For 3D reconstructions, about 25 *z* sections were collected at 0.3 µm *z* intervals. Reconstructions were performed using Imaris 3.0.6 analysis software (Bitplane AG, Zurich Switzerland). The *en face* view of the immune synapse was obtained by an *x-z* projection of the 3D image at the cellular interface of the NK and target cells.

### Image Analysis

Clustering or accumulation of a receptor at the NK cell immune synapse was defined as follows. Obvious conjugates between NK cells and target cells were first selected on DIC images and then fluorescent images were acquired. Clustering of NK cell receptors at the intercellular contact zone was defined as an obvious increase (usually 2 fold or greater) of the fluorescence intensity compared to the rest of the membrane. Fluorescence intensities were analyzed over the entire NK cell membrane on single confocal sections with the “Profile” function of the Zeiss LSM 510 software. This function allows the user to mark a line on a fluorescence microscope image and the software will report the intensity of every fluorophore at every position along the line. We used this function to draw a line around the entire membrane of the NK cell, starting at an arbitrary point roughly opposite the position of the NK:target cell contact. The software produces a graph of fluorescence intensity for each fluorophore at each point along the line vs. the relative position of each point along the line.
